# Cell-mediated nanoparticle delivery systems: towards precision nanomedicine

**DOI:** 10.1007/s13346-024-01591-0

**Published:** 2024-04-13

**Authors:** Ruoyu Cheng, Shiqi Wang

**Affiliations:** Drug Research Program, Division of Pharmaceutical Chemistry and Technology, Faculty of Pharmacy, University of Helsinki, Helsinki, FI-00014 Finland

**Keywords:** Drug delivery, Nanoparticles, Cell hitchhiking, Cell-mediated drug delivery

## Abstract

Cell-mediated nanoparticle delivery systems (CMNDDs) utilize cells as carriers to deliver the drug-loaded nanoparticles. Unlike the traditional nanoparticle drug delivery approaches, CMNDDs take the advantages of cell characteristics, such as the homing capabilities of stem cells, inflammatory chemotaxis of neutrophils, prolonged blood circulation of red blood cells, and internalization of macrophages. Subsequently, CMNDDs can easily prolong the blood circulation, cross biological barriers, such as the blood-brain barrier and the bone marrow–blood barrier, and rapidly arrive at the diseased areas. Such advantageous properties make CMNDDs promising delivery candidates for precision targeting. In this review, we summarize the recent advances in CMNDDs fabrication and biomedical applications. Specifically, ligand-receptor interactions, non-covalent interactions, covalent interactions, and internalization are commonly applied in constructing CMNDDs in vitro. By hitchhiking cells, such as macrophages, red blood cells, monocytes, neutrophils, and platelets, nanoparticles can be internalized or attached to cells to construct CMNDDs in vivo. Then we highlight the recent application of CMNDDs in treating different diseases, such as cancer, central nervous system disorders, lung diseases, and cardiovascular diseases, with a brief discussion about challenges and future perspectives in the end.

**Graphical abstracts:**

**Figure:**
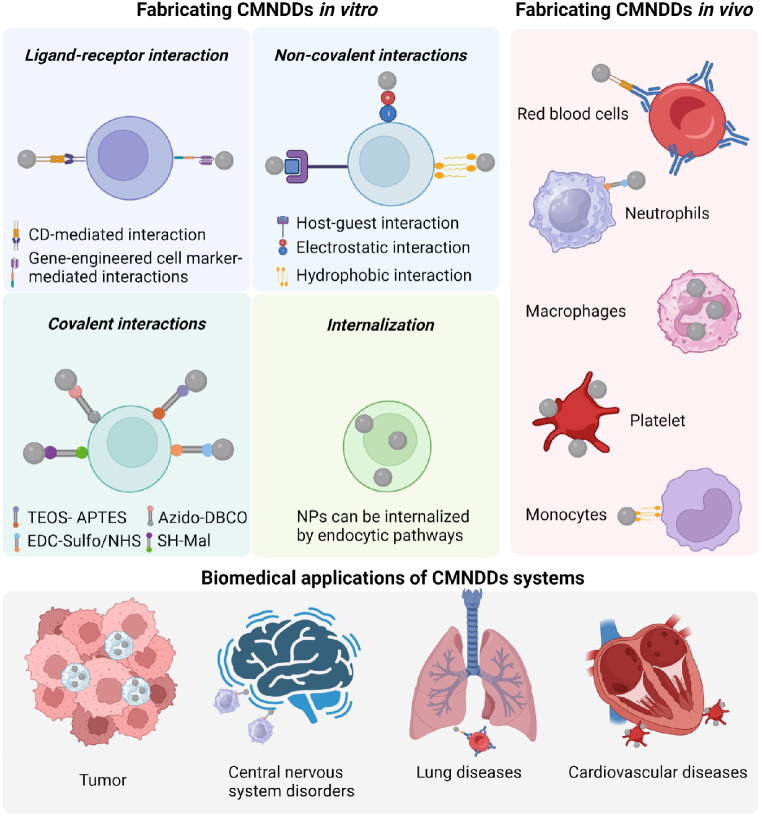
Cell-mediated nanoparticle delivery systems: a summary of their fabrication and biomedical applications

## Introduction

Precision nanomedicine is an emerging field, which offers unique opportunities for various biomedical applications. Particularly, nanoparticles (NPs) with precise targeting capabilities have been proposed, to overcome the biological barriers in the drug delivery, improve the drug concentration in specific tissues and cells, and extend the blood circulation duration of therapeutic reagents [1]. Various targeting strategies have been explored, such as active and passive targeting. Specifically, active targetabilities are associated with high-affinity interactions between NPs and targeted cells, such as antigen-antibody and receptor-ligand interactions [2]. However, the potential off-target and protein corona effects could influence the active targetabilities of NPs [3]. The passive targetabilities are associated with NPs’ physiochemical properties and tissues’ physiological characteristics. Specifically, the size, surficial zeta potential, morphology, and materials of NPs could regulate the biodistribution of NPs in vivo [4]. Additionally, the pathological or physiological properties of tissues, such as acidic tumor microenvironments and the scavenging effects of the reticuloendothelial system, also affect the biodistribution of NPs [5].

As the most well-known passive targetability, the enhanced permeability and retention (EPR) effect is attributed to the loose connection among endothelial cells, which allows the transportation of NPs from blood circulation to the tumor [6–8]. However, with a deeper understanding of NPs and cell behaviors, Warren Chan’s group proposed that the transportation of NPs into tumors was attributed to the transcytosis of vascular endothelial cells rather than EPR effects, which means that cells play an active role in the drug delivery, by selective NPs uptake and transport [9, 10]. This phenomenon suggests the possibility to develop cell-mediated NPs drug delivery systems (CMNDDs) in vivo. Different from traditional delivery strategies (active or passive targeting), CMNDDs can take advantage of different cellular characteristics, such as homologous homing, abundant surficial ligands, flexible morphologies, phagocytosis, differentiation, and metabolism, to maximize the drug delivery efficacy and minimize the potential side effects [11, 12]. Various cells can be utilized in designing CMNDDs, such as platelets, red blood cells, stem cells, and leukocytes, for prolonged blood circulation, locally increased drug concentration, and favorable biocompatibility in vivo [13, 14].

The concept of CMNDDs can be traced back to the development of cell-mediated drug delivery system loaded with free drugs [12]. In 1998, Flora et al. first encapsulated a new heterodinucleotide (consisting of both an anti-retroviral and an anti-herpetic drug, bound by a pyrophosphate bridge) into autologous erythrocytes [15]. To date, multiple methods have been developed to load drugs into cells with high loading efficiency, such as endocytosis, electroporation, and osmosis-based methods [16, 17]. For example, Magnani et al. successfully encapsulated dexamethasone-21-phosphate into human erythrocytes by osmosis in 2000 [18]. After several decades of investigation, some cell-based drug carriers have been evaluated in clinical trials [19]. For example, EryDel developed erythrocytes loaded with dexamethasone sodium phosphate for ataxia telangiectasia treatment (Clinical phase III, identifier: NCT03563053). However, the release behavior of the drugs loaded within cells cannot be precisely controlled. For example, the encapsulated drug cannot be responsively released under environmental stimuli, such as acidic environment, reactive oxygen species, etc [20]. Therefore, using NPs to protect drug from degradation or unexpected release in CMNDDs has been proposed.

**Scheme 1:**
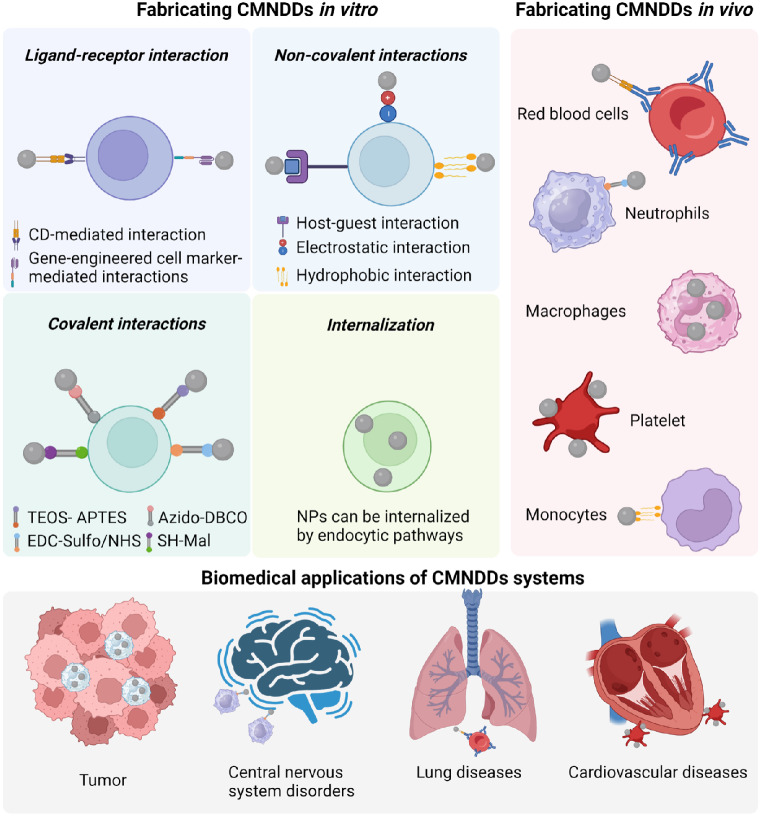
The scheme of fabricating CMNDDs in vitro and in vivo, and the representative biomedical applications of CMNDDs in different diseases. Created with BioRender.com

In this review, first we introduce how to fabricate CMNDDs in vitro and in vivo. In vitro fabrication means cells are engineered in vitro to load NPs and then infused to deliver drugs. In vivo fabrication means in vivo administrated NPs hitchhike cells and deliver drugs as CMNDDs. We analyze each fabrication strategy, followed by their biomedical applications in treating various diseases, as shown in Scheme 1. Finally, we provide perspectives on the future development of CMNDDs.

## Fabricating CMNDDs in vitro

The in vitro fabrication of NPs loaded cells requires co-engineering of NPs and cells. NPs should be biocompatible, metabolizable, and preferably with controlled drug release capability. As the biological host, cells should be able to interact with NPs, protect them in blood circulation and carry them across the biological barriers, such as the blood-brain barrier [21, 22]. The fact that living cells have dynamic metabolic activities and constantly interact with the NPs pose great challenges in constructing stable drug delivery systems. Thus, it is necessary to carefully select and evaluate the fabrication strategy. The most common strategy to incorporate NPs are internalization and surface modification *via* ligand-receptor interactions, non-covalent interactions, and covalent modifications [23, 24]. Each strategy has unique advantages and disadvantages, thus should be applied according to the cell types and NPs. A brief summary of the characteristics of these strategies is listed in Table 1.

**Table 1:** A brief summary of fabricating CMNDDs in vitro

Types of interaction	Approaches	Pros.	Cons.
Ligand-receptor interaction	Natural cell surface protein marker-mediated interaction [25–45]	Natural cell surface protein markers (such as CD) are widely expressed, and the fabrication is relatively easy.	The interaction is not specific enough, since some CD, such as CD45, CD3 are expressed on different cells, and the interaction could be influenced by the different conditions in vivo.
	Gene-engineered cell marker-mediated interactions [46]	The interaction is specific with high efficacy.	Gene-engineering is required for the design.
Other non-covalent interaction	Hydrophobic interaction [47]	NPs are anchored to the cell membrane by hydrophobic moieties.	The interaction may affect cell membrane integrity and cell function.
	Electrostatic interaction [31, 33, 48, 49]	Positively charged NPs attach to negatively changed cell membrane.	The interaction could be influenced by surrounding environments and the stability of the CMNDDs is concerning.
	Host-guest interaction [50]	This approach can be widely applied in fabricating CMNDDs regardless the types of cell carriers and NPs. The interaction is specific.	Chemical modification is needed on both cell carriers and NPs.
	Mechanical, osmotic and oxidative stress [51]	This approach can be generally used in fabricating CMNDDs.	The method could be harmful to cell membrane and cell function.
Covalent interaction	SH-Mal [52]	This approach is under the mild reaction conditions with high efficacy.	This approach is limited by the SH amount on cell surface and could affect cell function.
	Azido-DBCO [53]	This approach is under the mild reaction conditions with high efficacy.	This approach relies on the incorporation efficiency of azido sugars and is time-consuming.
	TEOS- APTES [54]	This approach provides silica layer to cells.	The silicification could influence the cell proliferation and cell viability.
	EDC-Sulfo/NHS [55]	This approach is under the mild reaction conditions with high efficacy.	This approach is limited by the amino amount on cell surface and could affect cell function.
	Schiff base [56]	This approach can be applied in various cell types.	Oxidation reaction is processed on the cell surface, which could influence the cell function.
	SPAAC [57]	This approach is under the mild reaction conditions with high efficacy	Chemical modification is needed on both cell carriers and NPs.
Internalization	The internalization of NPs by cell carries are mediated by endocytosis, pinocytosis, and phagocytosis [58–60].	This approach is not limited by either the cell types or the NPs; in addition, this approach is easy in practice.	The internalized NPs could be exocytosed by cell carriers, thus affecting the stability of CMNDDs. Additionally, the internalized NPs could regulate the cell behaviors, such as activation and differentiation. The NPs could inhibit the cell viability.

*Note* CD: cluster of differentiation, SH: thiol, Mal: maleimide, DBCO: dibenzocyclooctyne, TEOS: tetraethyl orthosilicate, APTES: 3-Aminopropyl) triethoxysilane, EDC: N-(3-dimethylaminopropyl)-N-ethylcarbodiimide hydrochloride, Sulfo-NHS: N-hydroxysulfosuccinimide sodium salt. SPAAC: strain-promoted azide-alkyne cycloaddition reaction

### Ligand-receptor interaction

As one of the main approaches for cell communication, ligand-receptor interactions are reliable and universal [27]. Generally, cell receptors are protein molecules embedded in the plasma membrane to provide the specific sites for ligand binding [61]. Ligand-receptor interactions can be generally divided into two types, natural cell surface protein markers-mediated interactions and gene-engineered cell marker-mediated interactions, which are applied in constructing cell-mediated NPs drug delivery systems [28, 29, 46].

**Fig. 1:**
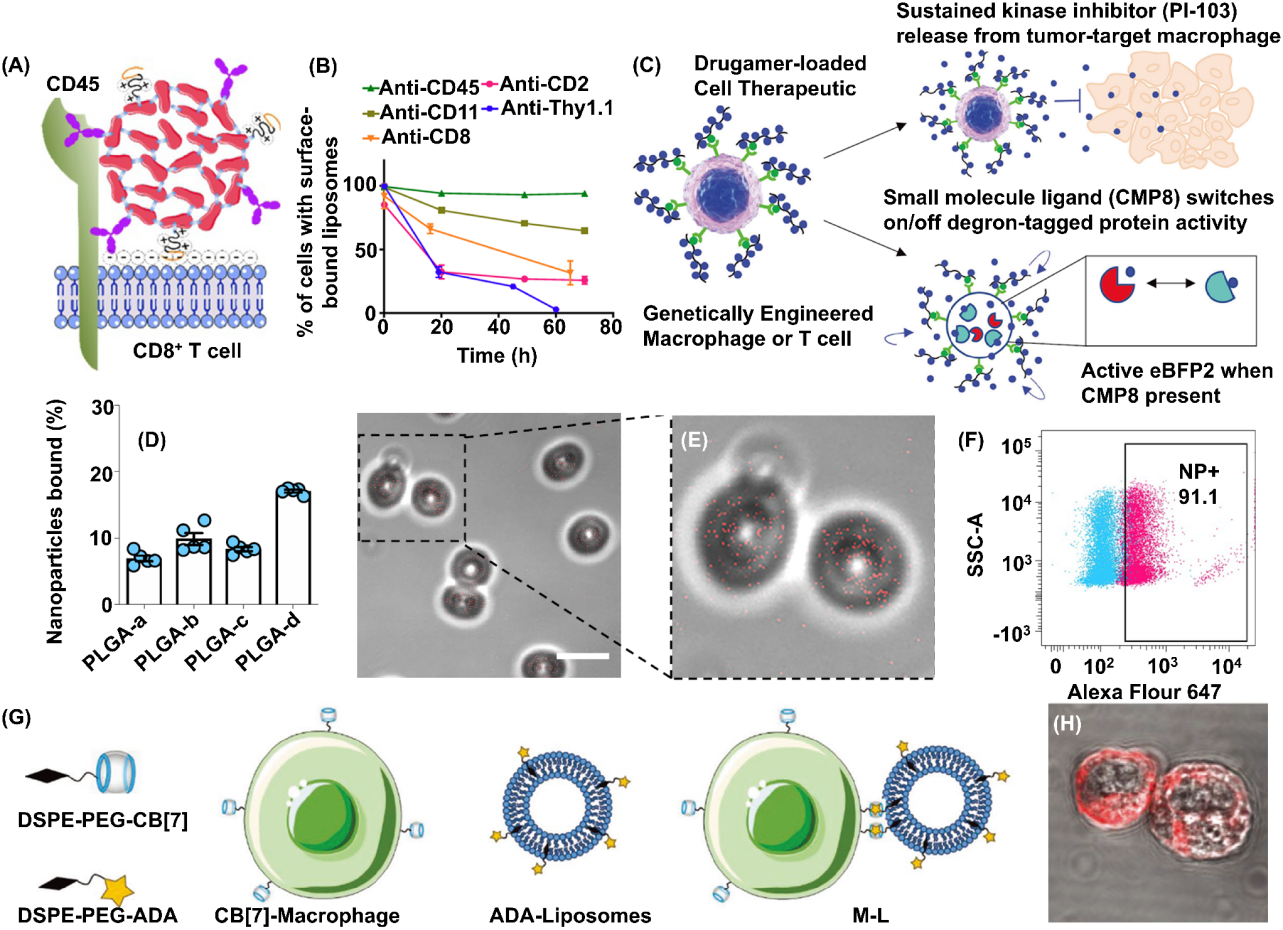
Fabricating CMNDDs by ligand-receptor interactions and other non-covalent interactions in vitro. (**A**). The scheme for surface modification of cytokine-nanogels to facilitate efficient and stable anchoring on T cell surfaces. (**B**) Flow cytometric analysis of biotinylated liposomes that were functionalized with the indicated monoclonal antibodies. **A** and **B** are preprinted with the permission from Springer Nature America Ref [26]. (**C**) The schematic mechanism of polymeric prodrug “drugamer” loaded cell therapeutics. Immune cells are engineered with a bioorthogonal and humanized scFv receptor that binds to polymeric prodrugs *via* a high affinity receptor–ligand binding reaction. **C** is preprinted with the permission from Wiley-VCH GmbH Ref [46]. (**D**) The binding efficiency of the PLGA-a (L: G ratio of 50:50, ester end), PLGA-b (L: G ratio of 50:50, acid end), PLGA-c (L: G ratio of 85:15, ester end) and PLGA-d (L: G ratio of 65:35, acid end) nanoparticles to erythrocytes. (**E**) The confocal laser scanning microscopy images of erythrocytes with NPs anchored on the surface. NPs was labelled with Alexa Fluor 647, Scale bar:10 μm. (**F**) Flow cytometry analysis of erythrocytes carrying Alexa Fluor 647-labelled NPs. The blue dots represent plain erythrocytes; the pink dots represent erythrocytes carrying NPs. **D-F** are preprinted with the permission from Springer Nature Ref [47]. (**G**) Schematic illustration of CB [7]-Macrophage, ADA-Liposome, and M-L (**H**) The confocal laser scanning microscopy images of CB [7]-Macrophages incubated with Dox loaded ADA-Liposome (Dox-L) for 4 h. **G** and **H** are preprinted with the permission from Wiley‐VCH GmbH Ref [50]

As one of the most common natural cell surface protein markers, cluster of differentiation (CD) protein profiles are commonly used as markers to identify cells. Different cell types express typical CDs on their surfaces, such as CD45 for leukocytes, CD3 for T cells, CD 4 for regular T cells, and CD8 for cytotoxic T cells [62]. These CDs can be specifically recognized by the corresponding antibodies, which could be used for anchoring NPs *via* ligand-receptor interactions. In addition, CD-mediated interaction could influence the binding and internalization efficacy of NPs, depending on the CD types. Irvine et al. synthesized various monoclonal-antibody-functionalized liposomes, including anti-CD2, anti-CD8, anti-CD11α, anti-CD90, and anti-CD45, to investigate the possibility of using CD and anti-CD interactions to anchor liposomes on T cell surface [26]. These monoclonal-antibody-functionalized liposomes can successfully anchor on the surface of T cells, and most of them can be internalized into the T cells within a few days. Interestingly, over 90% of anti-CD45 functionalized liposomes can retain on the T cell surface within 72 h, as shown in Fig. 1A and B. Additionally, the anchoring CD45 did not significantly inhibit T cell proliferation in response to anti-CD3/CD28 beads, suggesting that CD45 binding did not inhibit TCR signaling. Such liposomes anchored-T cells could be used to deliver interleukin (IL)-15 for tumor treatment.

CD-mediated interaction also regulates lymphocytes activation and function. Mitragotri et al. fabricated polymer micro-patches as natural killer (NK) cell engagers by CD45 and anti-CD45 induced cell attachment [29]. The polymer micro-patches comprised 70% poly (lactic-co-glycolic acid) (PLGA) and 30% PLGA-polyethylene glycol biotin to obtain free surficial PEG-Biotin sites. A streptavidin-conjugated CD45 antibody was modified to the polymer micro-patches *via* the biotin − streptavidin interaction; in addition, a range of antibodies and Fc-fusion proteins can be functionalized on the polymer micro-patches by the biotin − streptavidin interaction. Quantifying by flow cytometry, 46.6 ± 1.2% of NK-92 cells carried at least one micro-patch. Moreover, the attachment of micro-patches did not significantly influence the cell viability of NK-92 cells up to 96 h. However, the attached micro-patches could keep NK cells activated, by clustering and crosslinking different CD receptors. Such activation could enhance the in vivo antitumor efficacy of NK cells, without the need of cytokine coadministration.

Besides immune cells, the CD-mediated interaction can be applied in red blood cells (RBCs). Brenner et al. prepared the anti-CD modified liposomes by reacting azide-bearing liposomes with dibenzocyclooctyne (DBCO) functionalized monoclonal antibodies (anti-glycophorin A for binding red blood cells, anti-platelet endothelial cell adhesion molecule-1 and anti-intercellular adhesion molecule for binding endothelial) [25]. By incubating the liposomes with red blood cells (RBCs), the liposomes displayed specific, dose-dependent, saturable, and efficient loading onto mouse RBCs, achieving binding up to ~ 700 liposomes per RBC at maximal dose. When the liposomes were modified with 10% anti-glycophorin A and 90% anti-intercellular adhesion molecule, liposomes could bind to 99.7% of RBCs in suspension. Other CD molecules, such as CD11b [30], CD44 [31–33], CD73 [34], CD57 [35], CD161 [36, 37], and CD62 [38] mediated interactions between backpack and cells were also applied in constructing CMNDDs in vitro. Besides the CD-mediated interactions, immunoglobulin-antibody [39–41], biotin-avidin [41, 42], and biotin-neutravidin [43–45] mediated interactions can also be applied in constructing CMNDDs in vitro.

In addition to natural cell surface protein markers (CDs), gene-engineering could be used to generate specific cell markers for NP conjugation. Inspired by chimeric antigen receptor (CAR) T cells, Stayton et al. fabricated genetically engineered macrophages (GEMs) with AntiFl-𝜁, an artificial receptor which binds to a fluorescein ligand [46]. On the other side, ligand-tagged polymeric prodrugs (termed as “drugamers”) were synthesized by reversible addition–fragmentation chain-transfer (RAFT) polymerization with fluorescein (as ligands), PI-103 kinase inhibitor (as drugs), and poly (ethylene glycol) methyl ether methacrylate (PEGMA950), as shown in Fig. 1C. Due to the ligand-receptor interaction, 5 × 10^5^ AntiFl-𝜁 GEMs were approximately loaded with 3.8 µg PI-103 drugamer, equating to 0.5 µg PI-103. Compared to the untransduced (wild type) macrophages, AntiFl-𝜁 GEMs showed minimal changes to cell phenotype in terms of expressing CD40, CD80, CD86, human leukocyte antigen (HLA)-DR/DP/DQ, CD11b, PD-L1, CD163, CD206, and CD209. This CMNDDs could be used for glioblastoma inhibition, due to prolonged lifetime of PI-103 compared with free drugs. Furthermore, the gene-modified cell engineering method could be extended to T cells, to deliver small molecular drugs for protein activity regulation.

In summary, the specific interaction between ligands and the corresponding receptors can conjugate NPs with cell carriers in vitro without obvious cytotoxicity. Impressively, the modification of anti-CD45 on NPs can anchor NPs to those cells expressing CD45 and minimize the cellular internalization. However, as a broadly expressed surficial marker, CD45-mediated conjugation may encounter binding competition in vivo, i.e., the binding of anti-CD45 NPs with other cells. Furthermore, as CDs regulate cell activation and attachment, the conjugation with NPs unavoidably induces cell functional variations, which should be taken into consideration when designing such CMNDDs. In comparison, gene-engineered cell marker provides more specificity and flexibility when minimal interference on cell function is needed. However, the extra cell engineering step introduces transmembrane domain, surface tag, and an anti-fluorescein scFv, which requires exquisite design to maximize the expression of engineered markers, and to avoid potential changes to the cell phenotype.

### Other non-covalent interaction

Non-covalent interactions, such as hydrophobic interaction, electrostatic interaction, and host-guest interaction, are widely applied in constructing drug delivery systems due to the gentle fabrication process. During the anchoring process, electrostatic interactions, hydrophobic interactions, and hydrogen bonding can collaboratively participate in connecting NPs drug delivery systems with cells [63]. Mitragotri et al. prepared four different PLGA polymers regarding the lactic acid to glycolic acid (L: G) ratio and surface chemistry (acid or ester end) of PLGA [47]. Although different PLGA NPs could anchor to the erythrocytes, the PLGA NPs with a high L: G ratio and an acid end (PLGA-d) had high hydrophobicity and the ability to form hydrogen bonds, exhibiting the highest binding efficiency to erythrocytes, as shown in Fig. 1D–F.

Commonly, cell membranes exhibit negative surficial zeta potential, which can be utilized in interacting with cationic materials, such as polyethyleneimines [64]. Moreover, the cell membrane zeta potential can be regulated from negative to positive charges by pretreating cells with cationic polymers. Then, the positive-charged cell surface can be anchored with a negative-charged NPs drug delivery system. Guo et al. pretreated the tumor-homing macrophages (M𝜑s) with cationic cellulose, changing the cell surficial zeta potentials from − 14.7 to 16.0 mV [48]. By free-radical polymerization, N-(2-Hydroxyethyl) acrylamide (HEAA, as monomers), acrylic acid (AA, as monomers), N, N’-methylenebis (acrylamide) (MBA, as crosslinker), and ammonium persulfate (APS, as inhibitor) were synthesized to NPs. Then, the NPs were efficiently blocked by the iron-tannic acid (Fe^III^-TA) supramolecular networks with high doxorubicin (DOX) loading efficacy (191 mg/g). The NPs engineered M𝜑s (MAGN) can be constructed by rapidly mixing NPs with M𝜑s for 30 s; the anchoring effects could be attributed to the electrostatic interaction and hydrogen bonds. Notably, 71% of M𝜑s carried NPs, when incubated with 0.65 mg of NPs per 10^5^ cells. After normalization, M𝜑s can carry 88.6 µg of DOX per 10^5^ cells. Besides the cationic cellulose, electrostatic interactions between chitosan and hyaluronic acid are commonly used in constructing CMNDDs in vitro [31, 33, 49].

Host-guest interactions encompass the idea of molecular recognition and interactions through non-covalent bonding, which has raised dramatic attention since it was discovered in supramolecular chemistry [65]. Host-guest interactions play a critical role in many biological processes. In addition, it can be applied in designing CMNDDs. Typical macrocyclic host molecules, such as cyclodextrin, crown ether, and cucurbit [7]urils (CB [7]), and guest molecules, such as adamantane (ADA) and ferrocene, have been widely applied in host-guest interactions [66–69]. To fabricate CMNDDs by host-guest interactions, cell carriers need to be functionalized to incorporate either host or guest molecules. For example, as a widely used lipid molecule for membrane insertion, 1, 2-Distearoyl-sn-glycero-3-phosphoethanolamine-Poly (ethylene glycol) (DSPE-PEG) was first conjugated to CB [7] *via* thiol-ene click reaction between DSPE-PEG-SH and monoallyloxy cucurbiturils [50]. Then, the synthesized DSPE-PEG-CB was incubated with macrophages for 1.5 h to prepare the supramolecular macrophage (the macrophage cell membrane decorated with CB [7], 0.56 × 10^–6^ µmol CB per cell). Liposomes modified with ADA were prepared from soybean lecithin, cholesterol, and DSPE-PEG-ADA by film dispersion. Utilizing the host-guest interaction between CB [7] and ADA, the liposomes were connected with the macrophages after 4 h incubation in vitro, as shown in Fig. 1G. Although, the liposomes can be connected on the surface of macrophages even after 1 h incubation, 4 h incubation resulted in higher liposome stability on the surface of macrophages, as shown in as shown in Fig. 1H. Other researcher also applied mechanical, osmotic and oxidative stress to construct CMNDDs in vitro [51].

In summary, non-covalent interactions widely appear in fabricating cell-mediated NPs drug delivery systems in vitro. Compared with ligand-receptor interactions, hydrophobic interactions, electrostatic interactions, and host-guest interactions are moderated and reversible, which could benefit the loading and releasing of NPs on the surface of cell carriers. However, whether the binding effects between NPs and cells are strong enough to suffer the shear-force of blood circulation and complicated physiological environment should be comprehensively investigated.

### Covalent interaction

Fabricating CMNDDs by covalent interactions mean covalent bond formation between NPs and cell hosts [70]. Compared with receptor-ligand and non-covalent interactions, the affinity of covalent bonding is generally higher, due to the higher bond energy [71]. Thus, those NPs anchored to cell surface are more stable in the physiological environment. The reactions used to generate covalent interactions have to be mild, efficient and biocompatible with minimal interference on cell viability and function [72]. Examples of these reactions include thiol-maleimide (Mal), azido-dibenzocyclooctyne (DBCO), tetraethyl orthosilicate (TEOS)-(3-Aminopropyl) triethoxysilane (APTES), and N-(3-dimethylaminopropyl)-N-ethylcarbodiimide hydrochloride (EDC)-N-hydroxysulfosuccinimide sodium salt (Sulfo-NHS), etc. Typically, cell surface functional groups (thiols, azido, amine) react with the corresponding groups (Mal, DBCO and Sulfo-NHS) on NPs surface [73, 74].

**Fig. 2:**
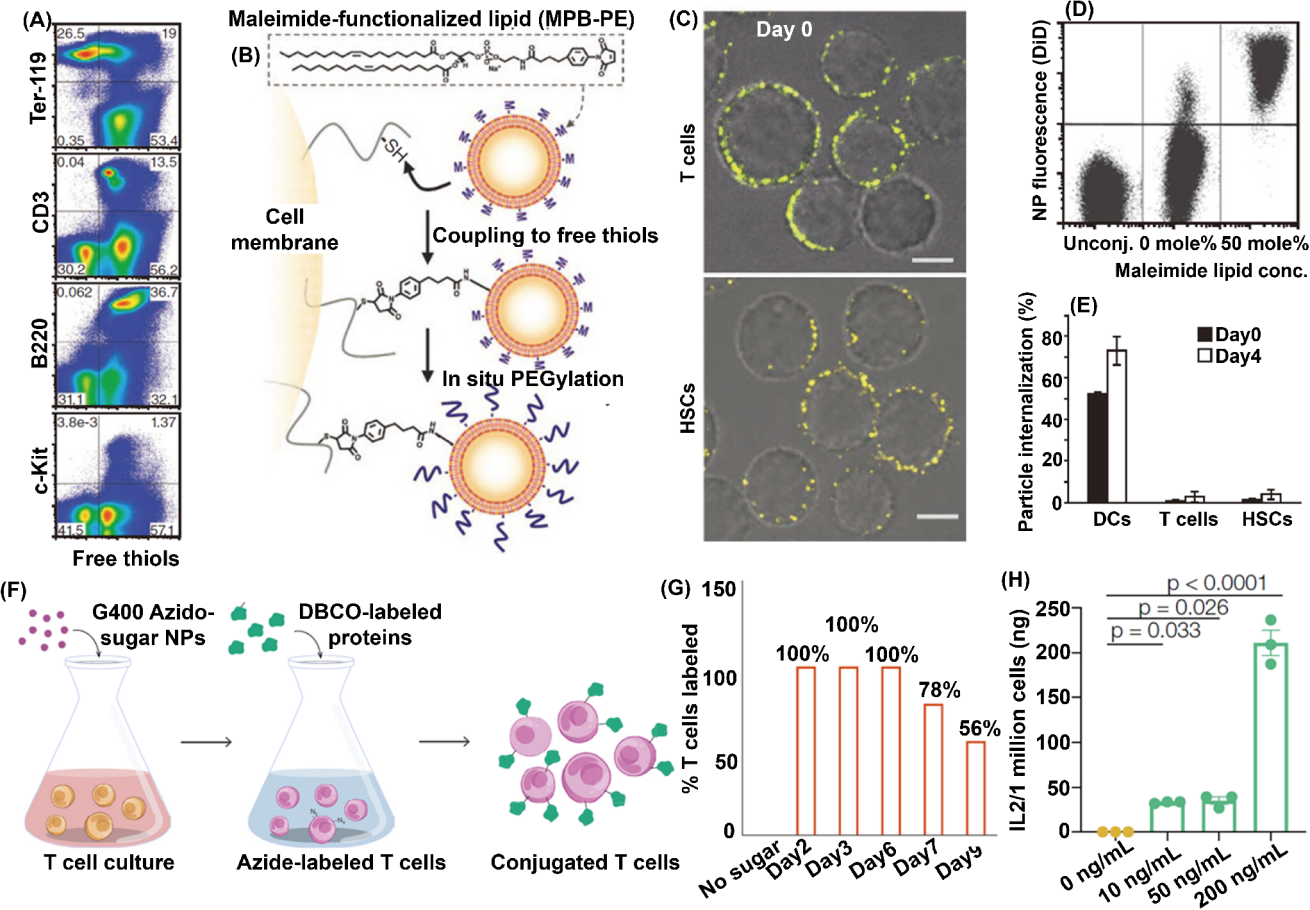
Fabricating CMNDDs by covalent interactions in vitro. (**A**) Flow cytometry analysis of cell surface thiols on mouse splenocytes detected by fluorophore-conjugated malemide co-staining with lineage-specific surface markers for erythrocytes (Ter-119), T cells (CD3), B cells (B220) and hematopoietic stem cells (c-Kit). (**B**) Schematic of maleimide-based conjugation to cell surface thiols. MPB-PE, 1,2-dioleoyl-sn-glycero-3-phosphoethanolamine-N-[4-(p-maleimidophenyl)butyramide]. (**C**) Confocal microscopy images of CD8^+^ effector T cells and lineage − Sca-1^+^c-Kit^+^ HSCs immediately after conjugation with fluorescent DiD-labeled multilamellar lipid nanoparticles. (**D**) Flow cytometry analysis of CD8^+^ T cells after incubation with DiD-labeled multilamellar lipid nanoparticles synthesized with or without maleimide-headgroup lipids. (**E**) Quantification of nanoparticle internalization. Immature dendritic cells (DCs), effector CD8^+^ T cells or HSCs were conjugated with carboxyfluorescein (CFSE)-tagged maleimide-bearing liposomes. **A-E** are preprinted with the permission from Springer Nature America, Inc., Ref [52]. (**F**) The schematic of metabolic labeling and cytokine conjugation of T cells with azido-sugar G400 NPs. Azido-sugar nanoparticles are directly added to T cell culture, enter T cells *via* endocytosis, and lead to presentation of azide group on T cell surfaces. After T cells are metabolically labeled, T cells are washed and DBCO-labeled proteins (e.g., cytokines) are directly added to produce conjugated T cells for downstream use. (**G**) The percentage of T cells with positive azide signal over time. T cells were treated with 200 µM G400 NP for 3 d, after which G400 NP in the medium was removed (day 0) and T cells were subsequently cultured free of G400 NP. (**H**) The enzyme-linked immunosorbent assay quantification of the amount of DBCO-IL-12 conjugated onto one million T cells at various DBCO-IL-12 concentrations. **F-H** are preprinted with the permission from PNAS [53]

To fabricate CMNDDs by thiol-Mal reaction, Irvine et al. first detected substantial amounts of free thiols on the surfaces of T cells, B cells, and hematopoietic stem cells (HSCs) but low amounts on RBCs, as shown in Fig. 2A [52]. As shown in Fig. 2B–E, NPs (size 100–300 nm), such as liposomes, multilamellar lipid nanoparticles and lipid-coated polymer nanoparticles, were modified with Mal and incubated with 3 × 10^6^ cell carriers such as, CD8^+^ T lymphocytes and lineage − Sca-1^+^c-Kit^+^ HSCs, at 37 °C for 30 min with gentle agitation every 10 min in vitro. Additionally, the unreacted Mal was quenched by incubating with 1 mg/mL thiol-terminated 2-kDa PEG at 37 °C for 30 min. Around 17.2 ± 8.7% of the total available T cell surface thiol groups can react with Mal-modified multilamellar lipid nanoparticles (140 ± 30 ~ 200-nm), which means that the attachment of 150 NPs with 200 nm in diameter would occlude only 3% of the surface of a typical 7-µm-diameter T cell.

In addition to thiols-Mal reaction, azido-DBCO reaction could be a general platform for constructing CMNDDs in vitro. As shown in Fig. 2F, Mooney et al. first metabolically labeled T cells with unnatural azido-sugar NPs, G400 NPs (G400 NP), a polymer of azido-sugar (*n* = 400) derived from N-azidoacetylmannosamine (Ac4ManAz) [53]. After 72 h of internalization, G400 NPs yield monomeric sugar-azide which was utilized in cellular metabolism and was integrated into membrane glycoproteins to provide azide groups on the T cell surface. The surficial modification was durable and efficient; T cells exhibited a positive azide signal after 3 days, and more than 50% of T cells maintained a positive azide signal for at least 9 days, as shown in Fig. 2G. Then various cytokines, such as interleukin (IL)-12, IL-21, and tumor necrosis factor (TNF)-α, were modified with DBCO, followed by incubating with azide-labeled T cells (1 × 10^6^ cell, 30 min at 4 °C) to conjugate the cytokines with T cells. This conjugation was dose-dependent, with higher concentrations of DBCO-cytokines resulting in a higher percentage of cell-surface cytokine. Around 200 ng cytokines could be modified on the surface of 1 × 10^6^ cells, as shown in Fig. 2H. The covalent interaction between DBCO and azide can also be potentially applied in conjugating NPs drug delivery systems with other immune cells. For example, Tang et al. prepared azide-modified macrophages, which were conjugated with gold NPs with DBCO linkers [75]. The gold NPs were also conjugated with a polyvalent spherical aptamer (AS1411) to enhance tumor targeting and recognition. The gold NPs-incorporated macrophages had an X-ray-induced activation due to reactive oxygen species generation by NPs.

Biosilicification *via* the covalent interactions between TEOS and APTES provide an interesting cell surficial modified strategy. Inspired by the biogenic silica formation, Mano et al. used chitosan-derived polymers as the organic template to induce silicification on human adipose-derived mesenchymal stem cells (hASCs) [54]. They first modified the polymeric backbone of chitosan with L-carnitine to induce quaternary amines and improve its solubility and cell attachment. Then hASCs were primed with L-carnitine-modified chitosan at 37 °C for 10 min. Afterwards, TEOS and APTES (molar ratio: 1:3) were added to trigger the silicification on the part surface of hASCs at 37 °C for 10 min. The hASCs holding a silica backpack exhibit enhanced cell survival in suspension conditions and can spread and acquire a more adherent phenotype.

As a classic approach to generate amide bonds, EDC/sulfo-NHS reaction was also utilized to fabricate the CMNDDs in vitro. Durymanov et al. prepare the peroxiredoxin-1 (Prx1)-loaded PLGA (acid terminated) microparticles, followed by activating the PLGA by EDC and Sulfo-NHS for 30 min at room temperature [55]. Then, the activating PLGA microparticles were incubated with NIH 3T3 fibroblasts at 37 °C for 1 h. By the EDC/sulfo-NHS coupling reaction, the covalent interaction was established between -COOH of PLGA microparticles and -NH_2_ of cells. Although the non-covalent interaction can also conjugate PLGA microparticles with 13.7 ± 1.8% NIH 3T3 fibroblasts, the covalent interaction increased the conjugated efficacy to 48.5 ± 1.0% IH 3T3 fibroblasts. Besides above-mentioned reactions, researcher also utilized Schiff base reaction [56] and strain-promoted azide-alkyne cycloaddition reaction [57] to construct the CMNDDs in vitro.

Unlike non-covalent interactions and ligand-receptor interactions, the covalent interaction is stable and irreversible, which may involve chemical modification on the cell surface to provide reacted sites for the conjugation of NPs. Metabolic labeling could be a general approach to modify cell surface with azido functional group that can be further reacted with DBCO-modified NPs through the bioorthogonal click reaction. By this approach, the CMNDDs could be easily fabricated *via* the covalent interaction in vitro.

### Internalization

Besides conjugating the cellular backpacks on the cell surface, cells can also internalize NPs and carry them to the destination [76]. As a normal cellular process, cells can internalize the surrounding materials by receptor-mediated endocytosis (e.g., clathrin-mediated endocytosis), pinocytosis, and phagocytosis [77]. After internalization of NPs into the cell carriers, whether these NPs would regulate the cell behaviors, such as differentiation and activation, should be comprehensively considered; in addition, whether the cellular internalization would influence the stimuli-responsiveness and pharmacology of NPs is a crucial concern.

**Fig. 3:**
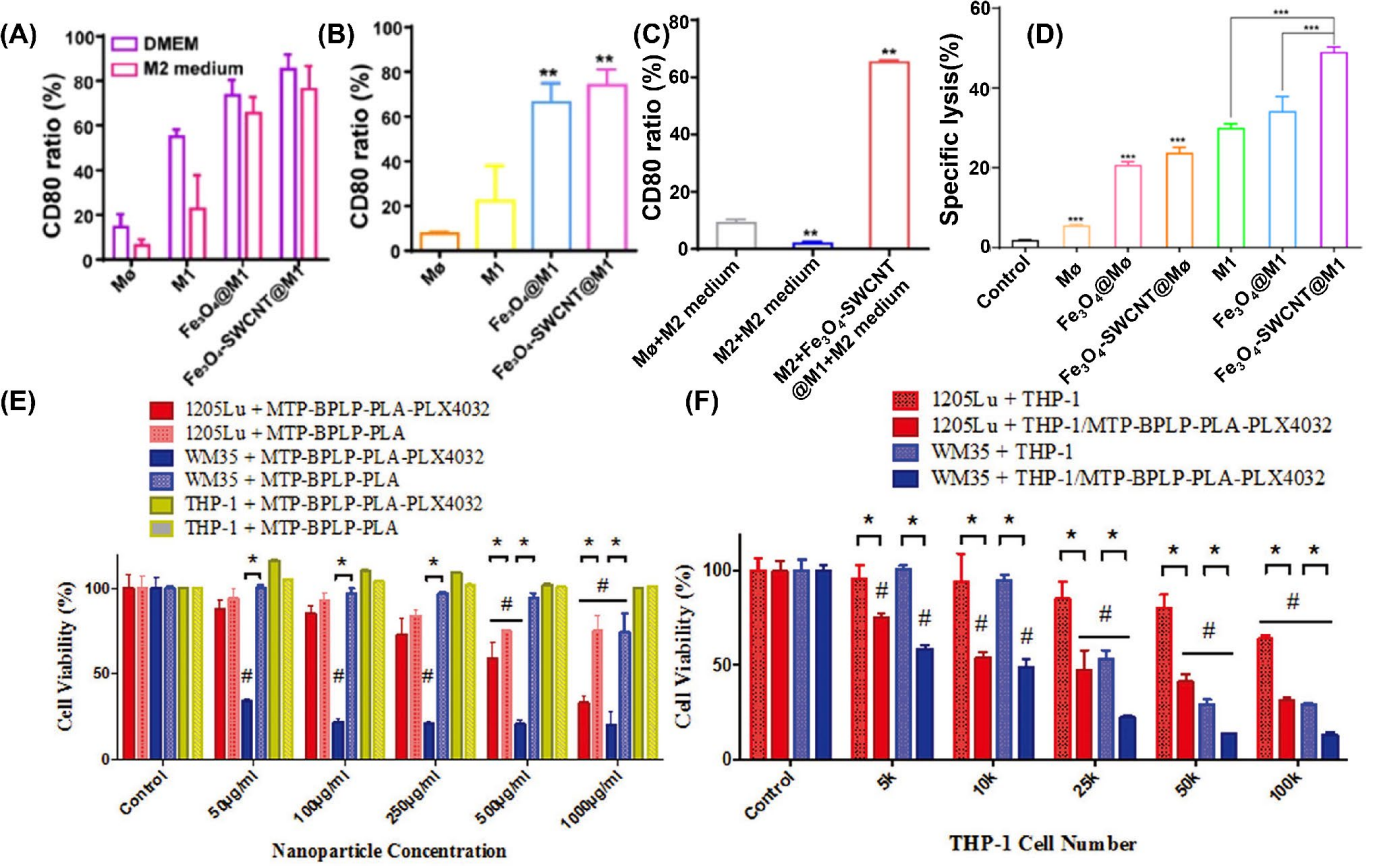
Fabricating CMNDDs by cellular internalization in vitro. (**A**) The change of CD80 expression in each group after 24 h incubation with DMEM medium and M2 culture medium. (**B**) The CD80 ratio in Mø+M2 medium, M1 + M2 medium, Fe_3_O_4_@M1 + M2 medium, Fe_3_O_4_-SWCNT@M1 + M2 medium group; (**C**) CD80 expression in freshly harvested M2 with different co-culture processes for 24 h; (**D**) The specific cleavage of 4T1 tumor cells in different groups by LDH method. **A-D** are preprinted with the permission from Elsevier Ref [58]. (**E**) Toxicity of MTP-BPLP-PLA and MTP-BPLP-PLA-PLX4032 nanoparticles to THP-1, WM35, and 1205Lu cells after 7 d of incubation. (**F**) THP-1-mediated nanoparticles delivery and drug release effects on melanoma cells with different THP-1 to melanoma cell (2000 cells per well) ratios after 7 d of incubation. #*p* < 0.01 compared to controls; *p < 0.01 between two groups. **E** and **F** are preprinted with the permission from Wiley-VCH GmbH Ref [59]

As professional phagocytes, macrophages are widely used as cell carriers to internalize NPs. Meanwhile, macrophages are also plastic immune cells that can respond to external stimuli and differentiate into distinct subtypes (M1 and M2 macrophages) [78, 79]. Therefore, whether the internalized NPs would regulate the differentiation of macrophages was explored by Li et al. First, the nano-sized ferroferric oxide/single wall carbon nanotubes composites (Fe_3_O_4_-SWCNT) were fabricated [58]. After incubating 50 µg/mL of Fe_3_O_4_-SWCNT with M1 macrophages for 4 h, the Fe_3_O_4_-SWCNT@M1 were successfully prepared. The Fe_3_O_4_-SWCNT@M1 can maintain the M1 subtypes (with the high expression of CD80) even in the M2 medium, as shown in Fig. 3A–C; in addition, the Fe_3_O_4_-SWCNT@M1 can specifically cleave 4T1 tumor cells by lactate dehydrogenase (LDH) method, as shown in Fig. 3D. After intravenous injection of Fe_3_O_4_-SWCNT@M1 (5 mg/kg with 3 × 10^6^ cells/mouse), the tumor progression was inhibited with activated tumor immune response.

Since the internalization of NPs into macrophages could regulate the differentiation of macrophages, whether the internalization of drug-loaded NPs into cell carriers would change the pharmacology of drug or cell functions is another concern. Yang et al. chose the PLX4032 as a model drug due to the specific inhibited effects in BRAF (V600E) mutation melanoma [59]. Then the PLX4032-PLA NPs were internalized into THP-1 cells to prepare the CMNDDs (THP-1 + MTP-BPLP-PLA-PLX4032). As shown in Fig. 3E, they found that pure NPs lacking the drug did not significantly reduce the THP-1 cell viability even at high nanoparticle concentrations (1000 µg/mL). But the pure NPs exhibited cytotoxicity on 1205Lu and WM35 melanomas at 500 µg/mL. In contrast, the PLX4032-loaed NPs can kill the 1205Lu and WM35 melanomas at 50 µg/mL, which indicates that drug released from nanoparticles were effective in killing melanoma cells. As shown in Fig. 3F, CMNDDs significantly decreased the viability of both 1205Lu and WM35 cells; even at the lowest THP-1 number (5000 cells/per well). Bare THP-1 cells without nanoparticles were served as control. Their results indicated that the PLX4032 can be released from CMNDDs with normal pharmacology that can be applied in specifically inhibiting melanoma cells.

After the internalization of NPs into cells, whether the NPs with responsiveness could still response to the stimulus should be carefully explored. Mitragotri et al. first integrated a photoactivated nitric oxide-releasing moiety (photoNORMs) with Nd^3+^-doped upconverting NPs (Nd-UCNPs), incorporating these nanoparticles into PLGA microparticles [60]. After 24 and 48 h of incubation, 100 mg/mL Nd-UCNPs-loaded PLGA microparticles were biosafe to bone marrow derived macrophages. After normalization, 263 µg microparticles or 0.667 mg manganese or 12.1 nano-equivalents of nitric oxide per 10^6^ bone marrow macrophages. Due to the photoNORMs, Nd-UCNPs can generate the nitric oxide (NO) under the stimulus of photo. Similarly, the bone marrow macrophages loaded with microparticles exhibited increased intracellular level of NO upon 794 nm laser exposure at 13.0 W/cm^2^ for 90 s, which indicates that the cell carriers did not influence the responsive properties of NPs.

In summary, the cell endocytic capability and physiochemical properties of NPs (morphology, diameter, surficial charge, and hydrophobicity) would influence the internalization and exocytosis of cells, resulting in the different NPs’ concentrations within cells. Moreover, the internalization of NPs could potentially regulate the differentiation and activation of some cells, such as macrophages, stem cells, and platelets. Consequently, the homing characteristics of cells would be changed according to the different cell subtypes. For example, after the internalization of NPs, the M0 macrophages could be differentiated to M1 macrophages that exhibit homing characteristics to inflammatory areas. Therefore, the internalization, exocytosis, differentiation, and activation of cells should be carefully considered during the fabrication of NPs-loaded cells by internalization strategy.

## Constructing the CMNDDs in vivo

Although most CMNDDs require in vitro cell engineering to anchor NPs on cell surfaces or internalized, CMNDDs can also be fabricated in vivo. Specifically, the drug-loaded NPs are first intravenously injected into the blood circulation, where the nanoparticles interact with circulating cells, such as macrophages, red blood cells, monocytes, neutrophils, and platelets [80, 81]. Although, there is still a debate on whether platelets are cells, this section will consider platelets as cells for the following content. After internalization or surface adhesion on these cells, NPs would be transported to specific tissues, such as tumors and infected areas, due to the tropism of cells [82]. This section will briefly introduce how to construct cell-mediated NPs drug delivery systems in vivo for different cell types, as shown in Table 2.

**Table 2:** A brief summary of fabricating CMNDDs in vivo

Cell types	Mediated markers	Applications
Neutrophils	Ly6G, Gr1, CD11b	Tumor [83–86], acute lung inflammation [87], inflamed skeletal muscle and ischemic heart [88]
Macrophages	Dectin-1, CD44	Tumor [89–92], acute lung injury [86, 93]
Monocytes	CD14	Tumor [94]
Red blood cells	No specific markers	Tumor [95, 96], acute lung disease [97], general drug delivering system [98]
Platelets	No specific markers	Thrombosis-associated diseases [99, 100], tumor [101–103]

### Neutrophils

Neutrophils are the most common leukocytes in the body, accounting for 50–70% of circulating leukocytes [104]. Additionally, adults generate around 100 billion neutrophils daily, and a similar number of senescent neutrophils must be replaced periodically to maintain homeostasis. Consequently, neutrophils have a relatively short half-life around 19 h [105]. When the senescence happens, the expression of C-X-C chemokine receptor type 4 (CXCR4) will re-elevate with the decreased expression of CXCR2 in neutrophils. With CXCR4/CXCL12 signals stimulation, the neutrophils migrate to bone marrow for apoptosis [106]. Additionally, neutrophils also play an essential role in the acute inflammation response. When tissue injury or pathogen invasion happens, neutrophils are rapidly activated in the bloodstream and are the first leukocytes to arrive at injured lesions, followed by the elimination of pathogens and initiation of inflammatory cascades [107]. Taking advantage of these characteristics, neutrophils are widely applied as a porter to deliver drug-loaded NPs to inflammatory tissues or bone marrow [87, 108, 109].

The morphology of NPs, such as sphericity or rod, could regulate neutrophil-mediated NPs drug delivery in vivo. Majouga et al. first prepared the PEGylated magnetic cubes and clusters NPs [83]. Then, these two NPs were intravenously injected into the established murine breast cancer (4T1) and colon cancer (CT26) models. Both NPs were captured mostly by intravascular neutrophils immediately after injection, and transmigration of NPs-bound neutrophils through the vessel wall was observed. The depletion of Ly6G and Gr1 (which are the markers for neutrophils), induced the decreased accumulation of NPs in the tumor, which confirmed neutrophils’ role as a carrier for targeting tumors. Moreover, the accumulation of shorter circulating NPs was more neutrophil-dependent than longer circulating NPs in tumors. Interestingly, the depletion of neutrophils almost totally blocked the delivery of cube NPs in 4T1 tumors. In contrast, the accumulation of cluster NPs was not completely blocked in 4T1 tumors. These results revealed that NPs with different morphology could be involved in various delivery mechanisms.

**Fig. 4:**
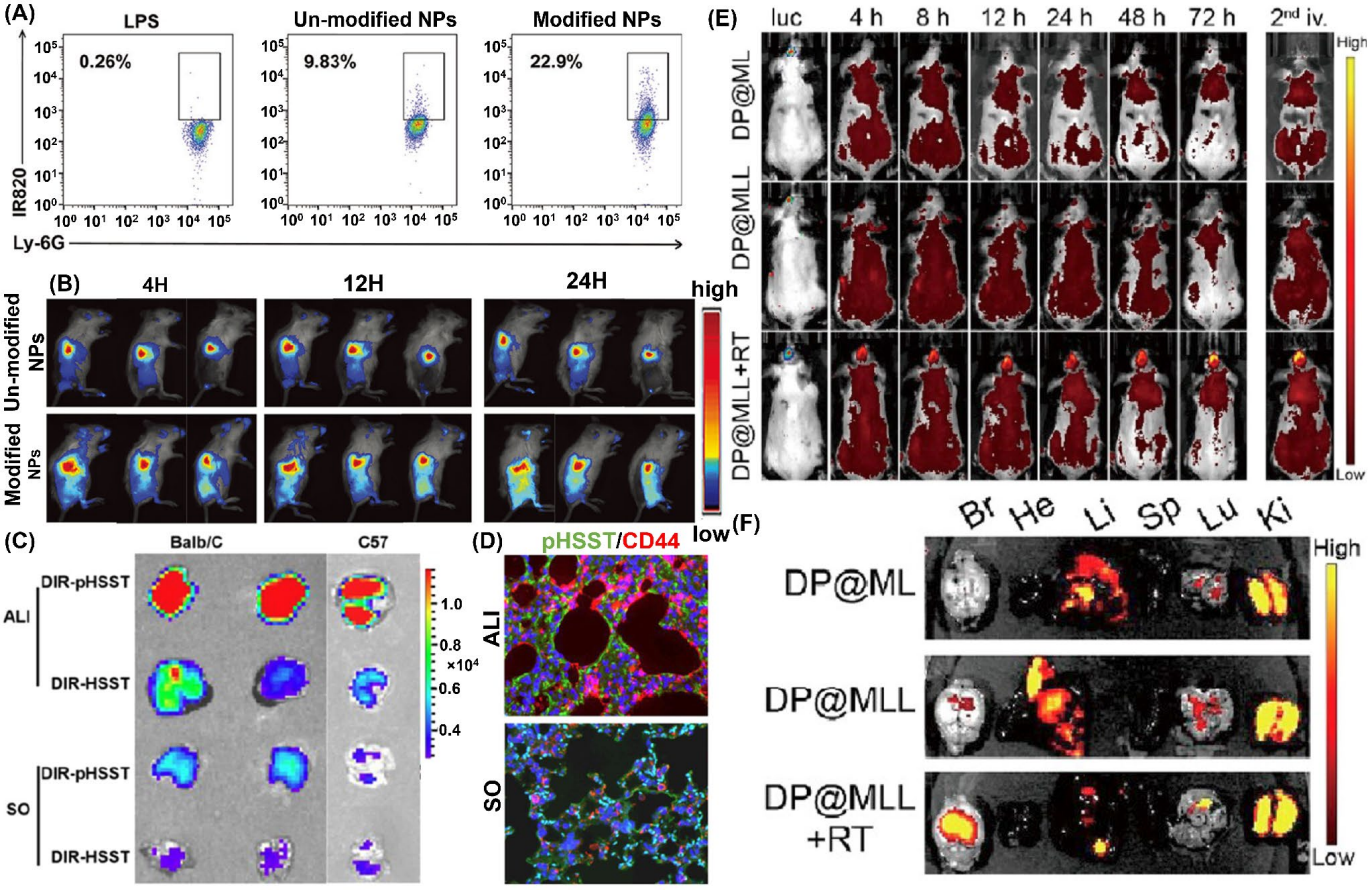
Fabricating CMNDDs by interacting with different cells in vivo. (**A**) Representative flow cytometry analysis of nanoparticles targeting neutrophils in the blood of postoperative mice. (**B**) In vivo imaging of postoperative mice 4, 12, and 24 h after un-modified NPs and modified NPs treatment. **A and B** are preprinted with the permission from Wiley-VCH GmbH Ref [84]. (**C**) Lung distribution of DIR-HSST and DIR-pHSST in acute lung injury (ALI) or sham-operated (SO) mice. (**D**) Immunofluorescence staining of lung sections indicated the signals of FITC-pHSST (Green) and CD44 (Red). **C** and **D** are preprinted with the permission from Elsevier Ref [93]. (**E**) Representative of PpIX fluorescence images after treatment with DP@ML (liposomes without modified peptides), DP@MLL (liposomes with modified peptides), or DP@MLL&RT (liposomes with modified peptides and pretreatment of radiotherapy) at different time points (*n* = 3). (**F**) PpIX fluorescence images of brain tumors and the major organs dissected from glioblastomas-bearing mice at 24 h after different treatments (*n* = 4). (Br, brain; He, heart; Li, liver; Sp, spleen; Lu, lung; Ki, kidney). **E** and **F** are preprinted with the permission from American Chemical Society Ref [94]

Regarding surface ligands, anti CD11b-modified NPs are favorably internalized by activated neutrophils. Lv et al. successfully integrated the anti-CD11b, decitabine, and IR820-conjugated bovine serum albumin into NPs [84]. Since the activated neutrophils expressed a high level of CD11b, the internalization of NPs in activated neutrophils increased from 9.8% (Unmodified) to 22.9% (Modified with anti-CD11b), as shown in Fig. 4A. The enrichment of anti CD11b-modified NPs in neutrophils are higher compared with other circulating cells such as monocytes, NK cells, or macrophages. Furthermore, more anti-CD11b-modified NPs accumulated at the tumor site than unmodified NPs from 4 to 24 h because of the rapid circulation of neutrophils in the blood and the inflammatory microenvironment in the tumor, as shown in Fig. 4B. According to the ex vivo tissue images, the relative fluorescence intensity of postoperative tumor sites in the anti-CD11b-modified NPs was 3.6 times higher than that in the unmodified group at 24 h.

### Macrophages

As professional phagocytes, macrophages express several phagocytic receptors, including scavenger receptors, to sense and recognize pathogen-associated molecular patterns, oxidized phospholipids, dead cells and internalize foreign materials in the blood circulation [110]. Macrophages are also plastic phagocytes with three major subtypes: M0, M1, and M2. These three subtypes of macrophages have distinctly different characteristics. M1 macrophages are highly involved in pro-inflammation, anti-tumor, and anti-infection, and M2 macrophages participate in anti-inflammation, tumor progression, and tissue regeneration. M0 macrophages are the resting state of macrophages [111]. The imbalanced polarization of macrophages is a significant driver for many diseases, such as pulmonary fibrosis. Inspired by the unique characteristics of macrophages, the researcher utilized macrophages as a carrier to deliver drug-loaded nanoparticles to specific tissues [90–92, 112].

Dectin-1 is abundantly expressed on the surface of macrophages and microfold cells (M cells). β-glucans can specifically interact with Dectin-1 by the receptor-ligand interaction [113]. Based on this interaction, Sung et al. designed a β-glucans-functionalized zinc–doxorubicin NPs (βGlus-ZnD NPs) that can first actively target M cells and then transcytosed across them, overcoming the intestinal epithelial barrier [89]. Afterward, βGlus-ZnD NPs were internalized into the endogenous resident intestinal macrophages *via* the interaction between Dection-1 and β-glucans. The endogenous macrophages can be excluded from immune reactions and respond to tumor-related chemokine/cytokine cues, transiting through lymphatic vessels, entering blood circulation, and eventually homing to the tumor tissues. Although most tumor-associated macrophages were M2 macrophages, the βGlus-ZnD NP-loaded macrophages can maintain the M1 subtype and remodel the immunosuppressive tumor microenvironment.

Besides the abundant expression of Dectin-1 on macrophages’ surfaces, the expression level of CD44 is also upregulated as an adhesion molecule. Hyaluronic acid can specifically recognize the CD44 [114]. By utilizing the interaction between CD44 and hyaluronic acid, You et al. integrated hyaluronic acid, α-tocopherol succinate, and pardaxin into NPs (denoted as HSST or pHSST with pardaxin peptide modified) [93]. In the established acute lung injury mouse model, macrophages were activated in response to numerous pro-inflammatory cytokines produced by immune cells, followed by recruiting to the inflamed tissue. While activation, macrophages highly expressed CD44 adhesion receptors and upregulated their phagocytic ability. Through specific recognition with the CD44 receptor, NPs were phagocytosed by the circulating macrophages after intravenous administration and then enriched in the inflamed lung tissue, which was about 7-fold higher than healthy mice, as shown in Fig. 4C and D. To prove whether the enriched NPs were associated with hitchhiking macrophages, chlorophosphonic acid liposomes were used to deplete macrophages in the acute lung injury mouse model. A significantly reduced accumulation of NPs can be observed in the lung because the depletion of macrophages reduces the hitchhiking effect of NPs.

### Monocytes

Monocytes are the most significant type of leukocyte in blood circulation. The lifespan of monocytes begins in the bone marrow. Once monocytes are matured, they enter the blood circulation to recognize and fight against foreign invaders [115]. They circulate in the bloodstream for about one to three days and then typically migrate into tissues throughout the body, where they differentiate into macrophages and dendritic cells [116]. Meanwhile, plenty of monocytes accumulate on some diseases, such as glioblastomas. As a primary approach in treating glioblastomas, radiotherapy can upregulate the expression of monocyte chemokine-1 (MCP-1)/C-C basal chemokine ligand 2 (CCL-2) in the tumor region, promoting the chemotaxis of monocytes [117]. Zhang et al. encapsulated doxorubicin hydrochloride in the matrix metalloproteinase 2 (MMP-2) responsive peptide-liposomes modified with lipoteichoic acid that can specifically bind to monocytes *via* the CD14 receptor [94]. After intravenous injection (4 to 72 h), liposomes (DP@MLL) exhibited enhanced accumulation in orthotropic GL261-bearing C57BL/6 mice compared to the liposomes non-modified with lipoteichoic acid (DP@ML). Moreover, mice pre-treated with radiotherapy (DP@MLL + RT) exhibited enriched NPs compared to those with un-pretreated radiotherapy (DP@MLL), as shown in Fig. 4E and F. Furthermore, significant fluorescence resonance energy transfer signals were observed in C57BL/6 mice injected with 1,1′-dioctadecyl-3,3,3′,3′-tetramethylindodicarbocyanine 4-chlorobenzenesulfonate salt (DiD)-labeled nanoparticles and 1,1-dioctadecyl-3,3,3,3-tetramethylindotricarbocyaine iodide (DiR)-labeled mouse peripheral blood monocytes. These results indicated that the NPs conjugated to the monocytes and hitchhiked to the brain tumor *via* the chemotaxis of monocytes.

### Red blood cells

Approximately 2.4 million new erythrocytes are produced per second in human adults. Red blood cells are developed in the bone marrow and enter the blood flow, circulating for about 100–120 days (each circulation takes about 60 s) before macrophages recycle their components [96, 118, 119]. Additionally, red blood cells lack cell nuclei and organelles, making them a promising drug delivery platform [97, 98, 120]. According to literature, the tertiary amine oxide (TAO)-containing zwitterionic polymer, poly (2- (N-oxide-N, N-diethylamino) ethyl methacrylate) (OPDEA) can hitchhike red blood cells to prolong blood circulation, and easily detach from the cells and bind to the luminal surface of endothelial cells, subsequently stimulating endocytosis and inducing adsorptive-mediated transcytosis of tumor endothelial cells for effective tumor extravasation and penetration. Shen et al. constructed chemotherapeutic nanodrug (SC)-loaded liposomes with TAO modifications [95]. After intravenous injection, the SC-loaded liposomes had a higher blood drug concentration compared to the free SC within 8 h of post-injection. Moreover, both TAO-modified liposomes and unmodified liposomes exhibited similar biodistribution at 0.5 h of post-injection. However, the TAO-modified liposomes exhibited a higher tumor accumulation and a low liver uptake compared to the unmodified liposomes at 48 h of post-injection, which revealed the red blood cells-mediated liposome deliver to tumor tissues.

### Platelet

Similar to red blood cells, platelets do not have cell nucleus either. When a blood vessel is injured, platelets maintain hemostasis by adhering to the vascular endothelium, aggregating with other platelets, and initiating the coagulation cascade [121]. Platelets exert muscle-like actomyosin-mediated contraction, significantly decreasing the overall clot size while increasing clot stiffness by several orders of magnitude [122]. Inspired by the characteristics of platelets, researcher fabricated various platelet-based CMNDDs [100–103]. Lam et al. integrated dextran, poly L-lysine, poly L-glutamic acid, and fibrinogen into polyelectrolyte multilayer (PEM) capsules [99]. Once the PEM capsules were intravenously injected, the host’s platelets bind to the surface of PEM capsules *via* the interaction between fibrinogen and αIIbβ3 integrin, forming platelet hybridized PEM capsule. The hybridized platelets can remain quiescent during circulation in normal physiological blood vessels. When the platelet hybridized PEM capsules find an injured blood vessel, the platelets activate *via* exposure to clotting activators. The activated hybridized platelets adhere to the platelet hybridized PEM capsule, exposing collagen, the platelet plug, or forming fibrin network − vehicle targeting. Subsequent integration into the fibrin network and resultant platelet contraction ruptures the platelet hybridized PEM capsule and releases the drug locally to abate bleeding.

Using various features of different cells, such as the inflammatory chemotaxis of neuropils, long circulation of RBCs, and aggregating properties of platelets, CMNDDs can be multi-functional and bionic. However, the cellular efflux, biocompatibility of NPs, internalized efficacy of NPs, and specificity of NPs, should be considered in fabricating the CMNDDs in vivo. Additionally, whether the loading-NPs could induce the differentiation of cells or change the biological functions of cells should also be checked.

## Biomedical applications of CMNDDs systems

Commonly, diseases are associated with changes in cell behaviors, such as metabolic patterns, expression of surface receptors, secretion of cytokines, etc [123]. Consequently, these abnormal cell behaviors induce different microenvironments and cell infiltration of diseases. In this section, we will briefly introduce the microenvironment and cell infiltration of some diseases, such as tumors, central nervous system disorders, cardiovascular diseases, lung diseases, and the application of CMNDDs in these diseases.

### Tumor

Tumor is associated with uncontrolled cell proliferation and immunosuppressive microenvironment [124]. Usually, the abnormally proliferating cells will be eliminated by the macrophages and cytotoxic T cells. However, cancer cells will develop various strategies to avoid elimination, such as do-not-eat-me signals, which could contribute to forming an immunosuppressive microenvironment [125]. Meanwhile, the low infiltration of lymphocytes, such as neutrophils, cytotoxic T cells, and NK cells, and the exhaustion of these lymphocytes further aggravate the immunosuppressive microenvironment [85, 86, 126].

**Fig. 5:**
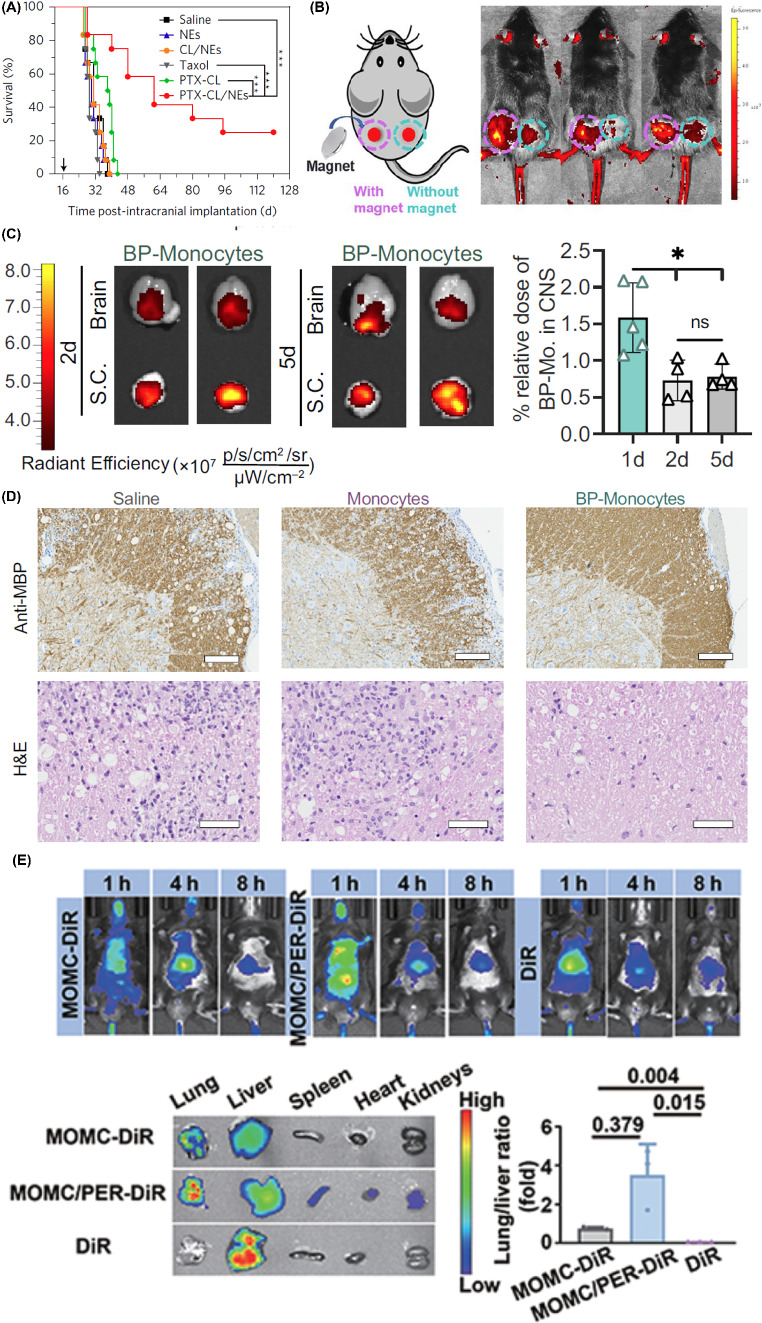
The biomedical applications of CMNDDs. (**A**) Survival curves of the surgically treated G422-bearing mice after intravenous administration of saline, the blank NEs (5 × 10^6^ cells/mouse), CL/NEs without PTX (5 × 10^6^ cells/mouse), Taxol (10 mg/kg PTX), PTX-CL (10 mg/kg PTX) and PTX-CL/NEs (5 × 10^6^ cells/mouse, equivalent to 5 mg/kg PTX) (*n* = 12 mice per group). Arrow indicates the time of the surgery. **A** is preprinted with the permission from Springer Nature Ref [86]. (**B**) Schematic diagram of a bilateral tumor model on the back of mouse, and fluorescence images of DiR-labeled NPs rearrangement in vivo under the magnetic field for 48 h photographed by in vivo imaging system. Pink circle, under magnetic field; Blue circle, without magnetic field. **B** is preprinted with the permission from Springer Nature Ref [128]. (**C**) Representative in vivo imaging images of brain and spinal cord displaying DiR 750 signal 2 day and 5 days after backpack-monocyte (BP-Mo) administration. Fluorescence quantification of relative dose accumulated in the CNS (cumulative brain and spinal cord signal); mean ± SD (*n* = 4). (**D**) Representative antimyelin basic protein (MBP) staining, revealing areas of demyelination, and hematoxylin and eosin (H&E) staining, revealing inflammatory infiltrates, for lumbar spinal cord sections of mice treated with Saline, Monocytes, and BP-Monocytes (*n* = 5). Anti-MBP scale bar represents 100 μm. H&E scale bar represents 50 μm. **C** and **D** is preprinted with the permission from PNAS Ref [131]. (**E**) In vivo fluorescence images of IPF mice intravenous injection with MOMC-DiR, MOMC/PER-DiR, and DiR (*n* = 3) and quantification of the in vivo retention profile (*n* = 3). **E** is preprinted with the permission from AAAS Ref [132]

Surgical tumor removal could induce local inflammation by releasing inflammatory factors, such as IL-8 and TNF-α, which would recruit the neutrophils to the inflamed tissues [127]. Taking advantage of these characteristics of neutrophils, Zhang et al. first prepared the paclitaxel-loaded cationic liposomes, followed by the internalization into neutrophils (18 µg paclitaxel/10^6^ neutrophils, denoted as PTX-CL/NEs) [86]. The in-situ glioma tumor model was established by intracranially implanting 1 × 10^5^ cells/mouse G422 or C6 cells into BALB/c mice (male, six-weeks old). At 16 days (G422) or 7 days (C6) after tumor implantation, the well-established tumors had formed, and the tumor-bearing mice were submitted for surgical resection of the tumor. Then, the PTX-CL/NEs (5 × 10^6^ cells/mouse) were intravenously injected in two mouse glioma surgical resection models. The PTX-CL/NEs exhibited the highest accumulation in the brain of surgically treated glioma-bearing mice than that of untreated glioma-bearing and sham-operated mice. The mice injected with PTX-CL/NEs exhibited 86-fold higher PTX concentration in the brain than the PTX-CL. Additionally, the PTX concentration arrived at the peak value (over 5 µg/g, PTX /brain weight) after 24 h of injection, then gradually decreased to lower than 2.5 µg/g at 72 h. Finally, the PTX was eliminated from brain after 120 h of injection. Consequently, the most extended 50% survival rate was observed in the PTX-CL/NEs groups (61 days) compared to the 38 days for PTX-CL, as shown in Fig. 5A. The neutrophil-mediated drug delivery system can recognize the postoperative inflammatory signals, such as IL-8 and CXCL1/KC, and deliver the chemotherapeutics to the infiltrating cancer cells spontaneously and on demand.

Since the therapeutic efficacy of adoptive cell therapy on solid tumors was greatly hindered by the insufficient tumor-infiltration of cytotoxic CD8^+^ T cells, You et al. anchored a dual-binding NPs platform consisting of PEG-Mal, HA, and Fe_3_O_4_ on the surface of T cells *via* the Michael addition reaction between the Mal (NPs) and the sulfhydryl groups (T cells) [128]. Guiding by the external magnetic field, the magnetic iron oxide NPs-anchored T cells migrated to the solid tumor, where the CD44 was highly expressed by the tumor cells, the average fluorescence intensity of the magnet-placed side was 2.3 times higher than that of the non-magnet side, as shown in Fig. 5B. In the E.G7-OVA tumor model, the tumors in the NPs-anchored T cells group were smallest, demonstrating a distinct inhibitory effect (decreased by 105%) on solid tumors compared with standard adoptive T cell therapy.

NK cell therapy, one of several immune-based therapeutic strategies, has been successful against liquid tumors, such as lymphomas and leukemias [129]. Encouraged by the anti-tumor capability of NK cells, Choi et al. conjugated NK cells with Sonazoid-microbubbles (NK-Sona) modified with human CD56 antibody for the real-time imaging and therapy of liver tumors [130]. The tumor model was established by subcutaneously injected with 1 × 10^7^ A549 cells on BALB/c nude mice (male, six-weeks old). After 3 weeks, 2.0 × 10^6^ NK cells, 2.0 × 10^6^ NK-Sona cells, and PBS were injected intratumorally into the mice three times at 3-day intervals. After intratumoral injection, the NK-Sona could be clearly detected by ultrasonography in tumor-bearing mice. Additionally, the anti-tumor capabilities of NK-Sona were proved in the A549-bearing subcutaneous tumor model by decreasing the tumor volume from around 700 mm^3^ (PBS group) to 500 mm^3^ (NK-Sona group), which was similar to the therapeutic effect of NK cells.

### Central nervous system disorders

A group of neurological disorders that affect the structure or function of the brain or spinal cord is denoted as central nervous system disorders (CNS) [133]. These disorders could be caused by infections, autoimmune dysfunction, blood clots, age-related degeneration, cancer, and injury, which lead to broad symptoms and treatments [134]. Different types of cells, such as monocytes, mesenchymal stem cells, and neutrophils, can carry NPs and cross the blood-brain barriers to treat CNS disorders [135].

Multiple sclerosis is a currently incurable autoimmune disease with inflammation and demyelination, leading to progressive neurodegeneration. During the progress of multiple sclerosis, adaptive immune cells, such as myeloid cells, are essential for initiating and exacerbating multiple sclerosis. Mitragotri et al. fabricated three layers of backpack (BP) consisting of two PLGA/dexamethasone layers modified with CD45 F(ab′) and a layer of PVA/heparin/IL-4 [131]. After incubating at 37 °C for 0.5-1 h, 60.5% of monocytes were attached to at least one backpack. The experimental autoimmune encephalomyelitis (EAE) murine model was established on female C57BL/6 mice (9–14 weeks) using the EK-2110 kit (Hooke Laboratories). In the EAE murine model, 3 × 10^6^ monocytes or backpack-laden monocytes (BP-monocytes) were intravenously injected. After 24 h, 1.59% BP-monocytes infiltrated in the CNS compared with 0.96% of monocytes alone, and over 60% monocytes were detected on the liver compared with around 50% of BP-monocytes. Even after 5 days of intravenous injection, there were still BP-monocytes in the CNS, as shown in Fig. 5C. Additionally, reduced inflammatory immune cell infiltration was observed in mice treated with BP-monocytes, compared to treatment with monocytes alone or saline, according to the histopathology analyses of the lumbar spinal cord on day 25 as shown in Fig. 5D.

Neuroinflammatory reactions, such as microglial activation and leukocyte invasion, occur in the ischemic brains. During the reactions, neutrophils are the first and most inflammatory cells in the microvascular response to ischemic stroke, which could be attributed to the high reactive oxygen species (ROS) level in the ischemic brains [136]. Tang et al. prepared the T-TMP NPs consisting of tetramethylpyrazine (TMP)-loaded ROS-responsive PLGA NPs and cinnamyl-F-(D) L-F-(D) L-F peptides that could target the neutrophil formyl peptide receptor [137]. In the middle cerebral artery occlusion (MACO) mice model, the T-TMP NPs attached to the surface of neutrophils and migrated to the ischemic brain at 1 h of intravenous injection. Then, the concentration of T-TMP NPs arrived at the highest level at 24 h compared to the non-modified NPs and SHAM group, which means that the T-TMP was more likely to enter ischemic brain tissue through the blood–brain barrier, and T-TMP exhibited a higher brain targeting efficiency. Additionally, the brain water content was significantly reduced with treatment with T-TMP than that of the MACO mice.

Besides the neutrophils, MSCs are another remarkable therapeutic platform in treating ischemic brains due to the homing capability of MSCs to the inflammatory sites, crossing the damaged blood-brain barrier, differentiating into functional neurons with immune-regulated effects and neuroprotective functions [138]. Chen et al. prepared the TK-M/Lu by integrating the DBCO-PEG2k-TK-PCL3.5k (ROS sensitive thioketal bond denoted as TK) polymers and luteolin [139]. Then the TK-M/Lu was attached on the surface of azide-modified MSCs (MSC-TK-M/Lu) after 2 h of incubation. The MCAO was established on Male Sprague-Dawley rats (240–250 g) by inserting a silicone-coated nylon thread (0.34 ± 0.02 mm) into the middle cerebral artery from the ipsilateral external carotid artery for 90 min. Then, the thread was removed, and the external carotid artery was ligated. One hour after MCAO surgery, 2 × 10^6^ MSCs or MSC-TK-M/DiR were intravenously injected. After 24 h of injection, MSC-TK-M/DiR mainly distributed in liver and spleen, followed by lungs. The lung accumulation could be attributed to the relative larger size of MSCs (approximately 10 to 20 μm), leading to the trapped MSC-TK-M/DiR. After 48 h, the fluorescence intensity of MSC-TK-M/DiR in the injured region was 4.1 times that of TK-M/DiR micelle. Notably, the biodistribution behavior of MSCs was not significantly altered after the modification of TK-M/DiR. The neurological score of MCAO rats was determined as 3.6, indicating severe neurological impairment. The neurological score of MCAO rats treated with MSC-TK-M/Lu decreased to 1.4, showing the greatest neurological recovery.

### Lung diseases

Lung diseases are pathological conditions affecting the organs and tissues, making gas exchange difficult in air-breathing animals. Lung diseases can be divided into obstructive pulmonary disease and restrictive lung disease according to the physiology. Similar to inflammatory diseases, the infiltration of immune cells could be a therapeutic platform for treating defective lungs [87, 140].

Idiopathic pulmonary fibrosis (IPF) is a rapidly progressive respiratory disease associated with the overactivation of myofibroblast and the formation of scar, which could cause an irreversible decline in lung function. Therefore, blocking myofibroblast activation and inhibiting collagen I deposition could be therapeutic in treating IPF [132]. However, delivering drugs to the lung is challenging due to the instability and tolerability of type II alveolar epithelial cells (AEC II). Jiang et al. integrated PLGA-PEG-Mal, PLGA-PEG-c(RGDfc), peptide E5, astaxanthin, and trametinib into PER NPs [141]. *Via* the interaction between peptide E5 of the PER NPs and the CXCR4 on the monocyte-derived multipotent cell (MOMC), MOMC/PER was established within 2 h of co-culture. Additionally, the PER NPs can stick to the surface of the MOMC without internalization by the MOMC within 8 h. The MOMC/PER had a loading capacity of 4.75 µg of trametinib and 1.5 µg of astaxanthin/1 × 10^5^ cells. The IPF was established on C57BL/6J male mice by inhalation of bleomycin through endotracheal intubation (2 U/kg, 40 µL). Then, the mice were intravenously injected with MOMC/PER-DiR, MOMC-DiR, and free DiR. Because of the MOMC’s homing ability, MOMC/PER reached directly to the lungs, the DiR fluorescence intensity in the lungs was respectively 3.5- and 0.5-fold greater than that in the liver in the MOMC/PER-DiR and MOMC-DiR groups, as shown in Fig. 5E. Additionally, the MOMC/PER can release the PER NPs retargeting the injured AEC II after responding to the matrix metalloproteinase-2 (MMP-2) in IPF tissues.

Besides the spontaneous homing capability of cell carriers, the homing capability can be further enhanced by the CMNDDs. Wang et al. respectively modified manganese dioxide nanoparticles (MnO_2_ NPs) with host molecules (β-cyclodextrin) and guest molecules (amantadine, AD) [142]. Macrophage was sequentially treated with these two NPs to initiate intracellular aggregations of NPs *via* the host-guest interaction. The microparticles could minimize the premature efflux of NPs during systemic circulation. The acute pneumonia model was established on BALB/c mice by intratracheal injection of lipopolysaccharides (8 mg/kg). The biosafety of delivering systems were evaluated in both healthy and acute pneumonia BALB/c mice after the intravenous injection of 1 × 10^6^ NPs-loaded macrophages. In healthy mice, no significant damage in the major organs or abnormal hematological parameters was observed post-injection for 24, 48, or 72 h. In the acute pneumonia mice, the liver and kidney function biomarkers and hematological parameters were sustained in the normal range comparable to those of the health control mice. In the inflammatory lung, the MnO_2_ NPs can react with H_2_O_2_ to generate O_2_ for self-propelling, increasing the tissue infiltration of engineered macrophages. Furthermore, the curcumin loaded MnO_2_ NPs can also be applied in engineering macrophages, and macrophages can be polarized to the M2 type for local anti-inflammation. In the acute pneumonia mice, the self-propelling, motorized cells acted as living carriers for targeted drug delivery and deep tissue penetration in the inflammatory lungs.

### Cardiovascular diseases

Cardiovascular disease describes the diseases involving the heart or blood vessels, such as stroke, myocardial infarction, aortic aneurysms, etc. After the myocardial infarction, neutrophils first arrive in the injured tissues and recruit monocytes that further differentiate into inflammatory M1-type macrophages [143]. After 3 days, the population of M1-type macrophages arrived at the peak value, and the M2-type macrophages had a peak accumulation after 7 days [144]. Therefore, researcher try to utilize these immune cells as effective carriers to deliver therapeutic payloads [88].

Considering these characteristics of myocardial infraction, Santos et al. fabricated pH-responsive putrescine-modified acetalated dextran (Putre-AcDEX) NPs, followed by modifying with atrial natriuretic peptide (ANP) and lin-TT1 peptide (Putre-AcDEX-PEG-TT1-ANP NPs) [145]. First, the myocardial infarcted model was established on male Sprague-Dawley rats (6–8 weeks old) by permanent ligation of the left anterior descending coronary artery. Then, 100µL NPs were intravenously injected. One hour later, the NPs can rapidly hitchhike on M2-type macrophages in vivo, increasing NPs accumulation in the infarcted hearts at 7 days post-myocardial infarction, due to the interaction with inflammatory cells. Moreover, NPs loading with two pleiotropic cellular self-renewal promoting compounds (CHIR99021 and SB203580) induced a 4-fold increase in bromodeoxyuridine incorporation in primary cardiomyocytes compared to control.

## Conclusion and perspectives

CMNDDs have become promising candidate for targeting drug delivery and disease treatment due to their unique properties in drug loading and biological functions, such as excellent biocompatibility, low immunogenicity, prolonged blood circulation, tissue-specific homing capability, and the ability to cross biological barriers. Various cells, such as RBCs, stem cells, T cells, NK cells, macrophages, neutrophils, and platelets, have been applied to construct CMNDDs. Compared with NPs drug delivery systems, the CMNDDs have a unique in vivo fate, which is attributed to their self-powered biological properties and functions and the effect of various factors, such as loaded drugs and loading process, administration route, pathological environment, and body response, which result in different drug delivery efficiencies and therapeutic outcomes.

CMNDDs can be successfully prepared in vitro *via* ligand-receptor interactions, non-covalent interactions, covalent interactions, and internalization. Moreover, NPs can spontaneously attach or be internalized on circulating cells, such as macrophages, red blood cells, monocytes, neutrophils, and platelets, nanoparticles, to construct CMNDDs in vivo. Regardless of whether the CMNDDs is constructed in vitro or in vivo, the biocompatibility, the internalized efficacy, the surficial grafting efficiency, and the potential cellular efflux of NPs should be carefully investigated. Regarding grafting NPs on the surface of cells, the relationship between the surficial residual concentration of NPs and incubating duration should be explored. Additionally, whether the modification of NPs on the cell surface would influence the cell-to-cell contact should be evaluated. As for internalizing NPs into cells, the potential biological changes, such as activation and differentiation of cells, should be considered. In addition, whether the internalized NPs would be rapidly excreted from the cells should also be investigated.

Although CMNDDs attracted great attention from various researchers, there are still critical challenges for clinical applications. First, the source of cells is a potential issue. Currently, the cell sources can be broadly categorized into three groups based on their origin: autologous, allogeneic, and xenogeneic [146]. Due to the potential host immune rejection, xenogeneic cells are not ideal candidates. There are three commonly used autologous cells: bone marrow-derived HSCs, immune effector cells isolated from peripheral blood, and induced pluripotent stem cells (iPSCs). However, the quality of these cells is closely related to the patient’s health [147]. For example, the immune effector cells could already be exhausted in the patient, which cannot be used to fabricate CMNDDs. Allogeneic cells could be a fascinating solution, but immunosuppression regimens, response durability, and cell engineering should be considered when applying allogeneic cells as part of CMNDDs [148]. Additionally, the ethics-related challenges should be carefully considered during the fabrication of CMNDDs, especially for those CMNDDs consisting of allogeneic cells.

Even if CMNDDs could be successfully fabricated in vivo or in vitro, it is still challenging to precisely control the drug releases in the targeted tissues. Additionally, avoiding the unexpected payload release in the blood circulation is another concern. Not only the CMNDDs would suffer from these challenges, but the NPs are also facing dilemmas [149]. Furthermore, we assume that CMNDDs could migrate to the targeted tissues due to their unique cell behavior. However, the biodistribution of CMNDDs cannot be fully controlled, which could not always lead to the increased accumulation of CMNDDs in targeted tissues. Furthermore, it is not known whether the NP loading changes the life cycle of the host cells after the drug release. In most published literature, long-term cell fate study is missing, and the host response to the suddenly increased cell population is also missing, which needs further investigation in the future.

Thirdly, the reproducibility of CMNDDs could be a huge challenge due to individual differences. Even the cell source can be autologous, but the individuals could exhibit different health conditions, such as different neutrophil percentages at different times, which could influence the reproducibility of CMNDDs. Such reproducibility issues will pose great obstacles from regulatory perspectives. Additionally, the stability and storage condition of CMNDDs is another concern.

Overall, CMNDDs provide new ideas for drug delivery, NPs design, and applications. Although CMNDDs are still facing enormous challenges, with the continuously updated understanding of cells and NPs, CMNDDs could be a powerful approach for treating various diseases.

## Data Availability

Not applicable.
